# Exploring the Use of Entomopathogenic Nematodes and the Natural Products Derived from Their Symbiotic Bacteria to Control the Grapevine Moth, *Lobesia botrana* (Lepidoptera: Tortricidae)

**DOI:** 10.3390/insects12111033

**Published:** 2021-11-17

**Authors:** Ignacio Vicente-Díez, Rubén Blanco-Pérez, Maryam Chelkha, Miguel Puelles, Alicia Pou, Raquel Campos-Herrera

**Affiliations:** 1 Instituto de Ciencias de la Vid y del Vino (ICVV), Gobierno de La Rioja, CSIC, Universidad de La Rioja, Finca La Grajera Crta. Burgos Km. 6 Salida 13 Lo-20, 26007 Logroño, Spain; ignacio.vicente@icvv.es (I.V.-D.); ruben.blanco@icvv.es (R.B.-P.); maryamchelkha@gmail.com (M.C.); mpuelles@larioja.org (M.P.); apou@larioja.org (A.P.); 2 Research Team “Lombricidae, Improving Soil Productivity and Environment” (LAPSE), Ecole Normale Supérieure (E.N.S.), Centre Eau, Ressources Naturelles, Environnement et Développement Durable (CERNE2D), Mohammed V University, Avenue Mohamed Bel Hassan El Ouazzani, Takaddoum, Rabat BP 5118, Morocco

**Keywords:** cell-free supernatant, grape, *Heterorhabditis*, *Photorhabdus*, *Steinernema*, secondary metabolites, unfiltered ferment, *Xenorhabdus*

## Abstract

**Simple Summary:**

The European grapevine moth (EGVM) *Lobesia botrana* (Lepidoptera: Tortricidae) attacks vineyards in Europe, the Middle East, and North and South America. Global movement toward sustainable agriculture urges the development of environmentally friendly tools that can replace traditional pesticides. Entomopathogenic nematodes (EPNs) are well-known biological control agents against various arthropod pests. The EPNs act together with symbiotic bacteria that produce natural products with insecticidal potential. Novel formulations and application technology allow their application against aerial pests, including those associated with vineyards. This study investigated the viability of four EPN species and their corresponding bacteria derivates (unfiltered ferment, UF, or cell-free supernatant, CFS) against EGVM (larval and pupa instars). The results revealed that all EPN species killed various EGVM larval stages. Killing pupae required a higher number of IJs than controlling larvae. *Steinernema carpocapsae* registered the most promising results, killing ~50% L1 and >75% L3/L5 in 2 days. The use of the bacterial bioactive compounds achieved similar results, with UF registering higher activity than CFS. Overall, we demonstrated that both EPN and bacterial bioproducts have a great potential to control EGVM in sustainable viticulture. Further research in co-formulation with adjuvants is required to ensure their survival in the aboveground grapevine areas.

**Abstract:**

The European grapevine moth (EGVM) *Lobesia botrana* (Lepidoptera: Tortricidae) is a relevant pest in the Palearctic region vineyards and is present in the Americas. Their management using biological control agents and environmentally friendly biotechnical tools would reduce intensive pesticide use. The entomopathogenic nematodes (EPNs) in the families Steinernematidae and Heterorhabditidae are well-known virulent agents against arthropod pests thanks to symbiotic bacteria in the genera *Xenorhabdus* and *Photorhabdus* (respectively) that produce natural products with insecticidal potential. Novel technological advances allow field applications of EPNs and those bioactive compounds as powerful bio-tools against aerial insect pests. This study aimed to determine the viability of four EPN species (*Steinernema feltiae*, *S. carpocapsae*, *S. riojaense*, and *Heterorhabditis bacteriophora*) as biological control agents against EGVM larval instars (L1, L3, and L5) and pupae. Additionally, the bioactive compounds from their four symbiotic bacteria (*Xenorhabdus bovienii*, *X. nematophila*, *X. kozodoii*, and *Photorhabdus laumondii* subsp. *laumondii,* respectively) were tested as unfiltered ferment (UF) and cell-free supernatant (CFS) against the EGVM larval instars L1 and L3. All of the EPN species showed the capability of killing EGVM during the larval and pupal stages, particularly *S. carpocapsae* (mortalities of ~50% for L1 and >75% for L3 and L5 in only two days), followed by efficacy by *S. feltiae*. Similarly, the bacterial bioactive compounds produced higher larval mortality at three days against L1 (>90%) than L3 (~50%), making the application of UF more virulent than the application of CFS. Our findings indicate that both steinernematid species and their symbiotic bacterial bioactive compounds could be considered for a novel agro-technological approach to control *L. botrana* in vineyards. Further research into co-formulation with adjuvants is required to expand their viability when implemented for aboveground grapevine application.

## 1. Introduction

The vineyard agroecosystem is one of the main study perennial crops, covering 7.5 M ha worldwide [1]. Controlling the principal pests and diseases is crucial for maintaining qualitative and quantitative production standards [2]. Conventional viticulture continues to be the most pesticide-consuming agricultural system even though worldwide interest in organic farming has increased significantly since the last decade [3]. Organic wine production aims at producing high-quality grapes and wines while minimizing the use of inputs and improving environmental care. The control of pests [4,5] and diseases [6] needs new biotechnological approaches that facilitate this possibility.

Even the accepted mean use of synthetic insecticides in conventional viticulture, grapevine moths are severe damage agents for grapes worldwide, causing yield losses and quality reduction. *Lobesia botrana* Denis & Schiffernüller (Lepidoptera: Tortricidae), known as the European grapevine moth (EGVM), is a relevant pest in European and the Middle Eastern vineyards. Current studies have reported EGVM as a new grape pest in the Americas: in Chile (2008) and Argentina (2009) [7,8] and in California (2010) [9]. Furthermore, global warming could have two critical collateral effects on the management of this pest. First, all of the suitable areas for *Vitis vinifera* are at risk of EGVM pest presence [10,11]. On the other hand, warming-derived phenological shifts imply a higher impact of first-generation EGVM and increased voltinism [12,13], increasing the land range EGVM’s damaging effects. The first larval generation of the season usually attacks inflorescence, while later generations cause damage to the fruits. In addition to direct damage on berries, pest occurrences result in disease due to the interconnected relationships in the entire agroecosystem. For example, the presence of larvae encourages bunch rot development (causal agents being *Aspergillus*, *Alternaria*, *Rhizopus*, *Cladosporium*, *Penicillium*, and *Botrytis*), which results in severe qualitative and quantitative damages [2,9,14].

Due to the mentioned progressive EGVM expansion and all of the damage that can they can cause to vineyards by grey mold induction [10], the interest in controlling EGVM is more pressing than ever [15]. Different effective measures to manage EGVM exist based on classical biological control methods and on the use of eco-friendly biotechnical tools (Table 1). For example, *Trichogramma* spp. is a natural enemy of EGVM due to its parasitic eggs [16], and *Bacillus thuringiensis* (*Bt*) is a well-known effective bio-insecticidal bacteria [6,17]. In addition, *Bt* produces several active compounds that are associated with pests and disease control, such as zwittermicin A and acyl homoserien lactonase [18]. Moreover, in organic viticulture, the pheromone-mediated mating disruption (MD) can be used against EGVM [19,20]. This environmentally friendly technique, which uses non-target effects, employs high doses of the pest´s synthetic sex pheromone in vineyard plots to interfere with its reproduction. Even though MD has some handicaps such as socio-economic challenges that hinder the introduction of this approach among the other grower tools, it requires pretreatment with conventional insecticides that have been endorsed by IPM regulations and needs at least a 5 ha extension to be effective [20].

The implementation of good integrated pest management (IPM) that enhances the presence of biological control agents could facilitate an overlap between pests and their natural enemies. In addition, expanding current biotechnological control tools is needed, especially in organic crop management (Table 1). In this context, entomopathogenic nematodes (EPNs) are good candidates with probed virulence toward numerous arthropod pests [25,26]. Their non-feeding, free-living infective juvenile (IJ) stage can penetrate the hemocoel of the host and can release a mutualistic enteric γ-Proteobacteria (genera *Xenorhabdus* for steinernematids and *Photorhabdus* for heterorhabditids). The Phase-I symbiotic bacteria overcome the host’s immune response through the secretion of a wide variety of biologically active compounds. These natural products (NPs) have broad-spectrum activity that can result in two effects: (1) toxicity effects (insecticidal, fungicidal, antibiotic activity) [27] and (2) deterrent effects that allow the EPN to protect the cadaver by deterring opportunistic and scavenger organisms [28]. The bacterial growth increases exponentially, resulting in the death of the arthropod by cause of septicaemia within 48–72 h of infection. Inside the cadaver, the EPNs feed on their partner bacteria and the degraded host tissues. Due to the resources being depleted, second-stage juveniles develop to the IJ stage, incorporate some of the symbiotic bacteria, and exit the insect cadaver by the thousands into the soil to start a new cycle [26,29,30,31].

In the context of modern viticulture, we considered that the use of EPNs as well as the use of the bioactive compounds that are obtained by their symbiotic bacteria could be an additional alternative to chemical treatments (Table 1) [32]. Although one of the limitations for the use of the EPN against the EGVM is their main distribution in the aboveground part of the vineyard, the current biotechnological improvements in the aerial application of EPNs has broadened the range of target pests, including this tortricid species [33]. Previous studies have shown the compatibility of EPN aerial applications against various tortricid species such as *Cydia pomonella* (L.) and *Thaumatotibia leucotreta* (Meyrick) (Lepidoptera: Tortricidae) [34,35,36,37,38]. Furthermore, a recent study has shown the high virulence of two EPN species (*S. yirgalemense* and *S. jeffreyense*) against tortricid larvae in the species *Lobesia vanillana* (De Joannis) (Lepidoptera: Tortricidae), a sporadic pest in vineyards in South Africa [39]. However, to date, there is no information about the compatibility of EPNs against the widespread EGVM. In addition, it is still unknown whether the natural products produced by their symbiotic bacteria can control EGVM. This study aimed to explore their use against various larval instars of *L. botrana* (L1, L3, and L5) and their pupal stage. This study settles the basis for the long-term goal of developing new bio-tools that provide an efficient alternative for the integrated management of EGVM.

## 2. Materials and Methods

### 2.1. Insects and Nematode Rearing

The EGVM population used to test EPNs was obtained from the Public University of Navarra (Spain), but for the test with natural products generated by the bacterial symbionts, we had to employ new specimens (because of the COVID-19 lockdown), which were supplied by Dra. Ally Harari (Department of Entomology, Volcani Center, Israel). All individuals were reared in an environmentally controlled chamber at 22 ± 1 °C and 60 ± 10% RH, with 16:8 (L:D) photoperiods, at the Institute of Grapevine and Wine Sciences (ICVV, Logroño, La Rioja, Spain). Under these conditions, we placed 20–30 adults into one transparent truncated conical cup with one piece of honey-soaked cotton (1:10 water-diluted) as a source of nutrients. Every 2–3 days, the eggs that had been laid all over the plastic surface were collected and combined from all of the adult cups in the rearing boxes with filter paper on the bottom and pieces of a semisynthetic diet (Supplementary Material, Table S1). We checked larval growth 2–3 days per week, adding food as needed while they completed their five larval instars. Lastly, we removed the pupae in order to start the ovipositional protocol with new adults. The same larval cohort age was employed for each experimental trial.

The EPN populations that were evaluated, *Steinernema feltiae* RM-107, *S. carpocapsae* ALL, *S. riojaense* RM-30, and *Heterorhabditis bacteriophora* RM-102 (Table 2), were cultured in *Galleria mellonella* (Lepidoptera: Pyralidae) larvae, which had also been reared at ICVV in an environmentally controlled chamber at 28 ± 1 °C and 20 ± 10% RH without a photoperiod and using an artificial diet (Supplementary Material, Table S2). The IJs were recovered in tap water upon emergence, stored at 12–14 °C, and used within two weeks of harvest.

### 2.2. Symbiotic Bacterial Isolation and Natural Products Generation

Three *Xenorhabdus* species (*X. bovienii*, *X. nematophila*, and *X. kozodoii*) and *Photorhabdus laumondii* subsp. *laumondii* were isolated from their respective mutualistic EPN species (Table 2), following the protocols of Vicente-Díez et al. (2021) [40]. Briefly, we first cleaned ~500 IJs of each EPN population by immersion in 5% NaClO for 2–5 min and then washing them with distilled water (three times) before bacterial extraction. Then, we mechanically disaggregated the IJs in a 50:50 (*v*/*v*) suspension of distilled water and nutritive broth (VWR, Chemicals, Barcelona, Spain) using sterile blue pestles (15 s) that had been assembled to a Kontes™ Pellet Pestle™ motor (DWK Life Sciences GmbH, Mainz, Germany). For each EPN species, we seeded 50 µL of this nematode–bacterium complex suspension on three Petri dishes with Nutrient Agar (NA, VWR, Dorset, UK), Bromothymol blue (Alfa Aesar, Kandel, Germany), 2,3,5-Triphenyl tetrazolium chloride (TTC, VWR, Chemicals, Barcelona, Spain) (NBTA plates), and Ampicillin (50 mg/mL) (PanReac AppliChem, ITW Reagents, Barcelona, Spain). After 48 h, we selected a colony in Phase I from the NBTA medium to generate pure bacterial cultures by further subculturing them in new NBTA plates. All of the bacterial strains were refreshed weekly into another NBTA plate, checking for purity based on morphology and color.

We obtained the bioactive compounds produced by *Xhenorhabdus* and *Photorhabdus* by inoculating single colonies of each bacterium into two Erlenmeyers with 250 mL of Tryptone Soya Broth (TSB) (VWR Chemicals, Barcelona, Spain). We incubated this culture at 150 rpm and 25 ± 2 °C in darkness for three days to obtain the unfiltered ferments (UF). Finally, we used one of the containers to generate cell-free supernatants (CFS). First, we centrifuged the bacterial suspension at 68.905× *g* (Thermo Scientific™ Sorvall LYNX 4000 Superspeed Centrifuge, Fisher Scientific SL, Madrid, Spain) for 20 min at 4 °C. Then, the supernatant was filtered through a 0.22 µm sterile pore filter [40]. An aliquot of this filtrate was cultured on NBTA plates in duplicate to verify the absence of bacteria. The pellet obtained after the centrifugation was also cultured in NBTA plates to check that the bacteria were still in Phase-I. The TSB used as controls were also filtrated to maintain all treatments under the same conditions.

### 2.3. Larvicidal and Pupicidal Assays

The larvicidal activity of IJs and bacterial products (UF and CFS) was tested against different EGVM larvae instars following the same methodology. We performed independent assays for each combination of EGVM larval stage and EPN/bacteria product. The experimental unit was a Petri dish (55 mm diam.) covered with one Whatman no.1 filter paper, with each containing five larvae of the corresponding instar and diet (to ensure food *ad libitum*, see details below). The dish was closed tightly with Parafilm and incubated in a growth chamber under controlled conditions (22 °C, 60% RH, and 16L:8O). Each treatment (EPNs, UF, CFS, and their corresponding controls) comprised six Petri dishes (30 insects per treatment in group of five per dish), and each experiment was performed twice (at different times) with freshly produced IJs, UF/CFS stocks, and insects. Larval mortality was checked daily for five days.

For the EPN assays against L1, L3, and L5 instars, we added ~1 cm^3^ of semisynthetic diet as a source of nutrients. Each EPN population was applied in a volume of 400 µL in a final concentration of 10 JIs/cm^2^ using distilled water in the control treatments. In addition, based on the preliminary results, we performed a lethal concentration (LC) response test against L5 instars for the most virulent EPN populations (*S. carpocapsae* and *S. feltiae*). In this case, the concentrations were 10, 5, 2, and 1 IJs/cm^2^ in a final volume of 400 µL (only distilled water for negative controls). All of the tests were conducted in the same controlled conditions as those reported before. On the other hand, we tested the toxicity of the natural products against the L1 and L3 instars. In this study, L5 was excluded because the larvae did not eat enough for any visible effect on the mortality to be observed. In the same experimental unit as the one described before, we replaced the semisynthetic diet with UF and CFS products that had been thickened with the addition of 0.1% agar bacteriologic (ITW reagents, Panreac, Barcelona, Spain) and supplied with 0.05% Methyl 4-hydroxybenzoate (Nipagina) (Sygma Aldrich, Barcelona, Spain) to avoid contamination in the diets. Specifically, we placed five hundred milligrams of each solidified medium diet in each Petri dish (filtered TSB was used for control treatments).

To test the pupacidal activity of the IJs, we employed two 24-multi-well trays (Corning, New York, NY, USA) per treatment using 12 interleaved wells per tray. In each selected well, we added 1 g of sterilized sand (pure sand, Vale do Lobo, Loulé, Portugal) and one EGVM pupa (no sexual dimorphism accounted). Immediately after, we inoculated 50 or 100 IJs in a final volume of 200 µL (only distilled water for negative controls). We checked how many moths had hatched daily for ten days. Each experiment was performed twice with freshly produced IJs, pupae, and subtracts.

### 2.4. Statistical Analysis

We ran general linear models (GLM) with a binomial distribution (logit-link function) for the pair treatment comparisons (control *versus* treatment) to test the impact of the IJ and bioactive compound (UF and CFS) virulence on the EGVM larval and pupal instars. We performed a Probit analysis to calculate the lethal concentration (LC) that could kill 50 and 90% of the population (LC_50_ and LC_90_) and the regression line slope. We performed all of the analyses with SPSS 25.0 (SPSS Statistics, SPSS Inc., Chicago, IL, USA), using *p* < 0.05 to assess the statistical differences. We used least-square means ± SE as descriptive statistics.

## 3. Results

### 3.1. Larvicidal Effect by Entomopathogenic Nematodes

**Figure 1:**
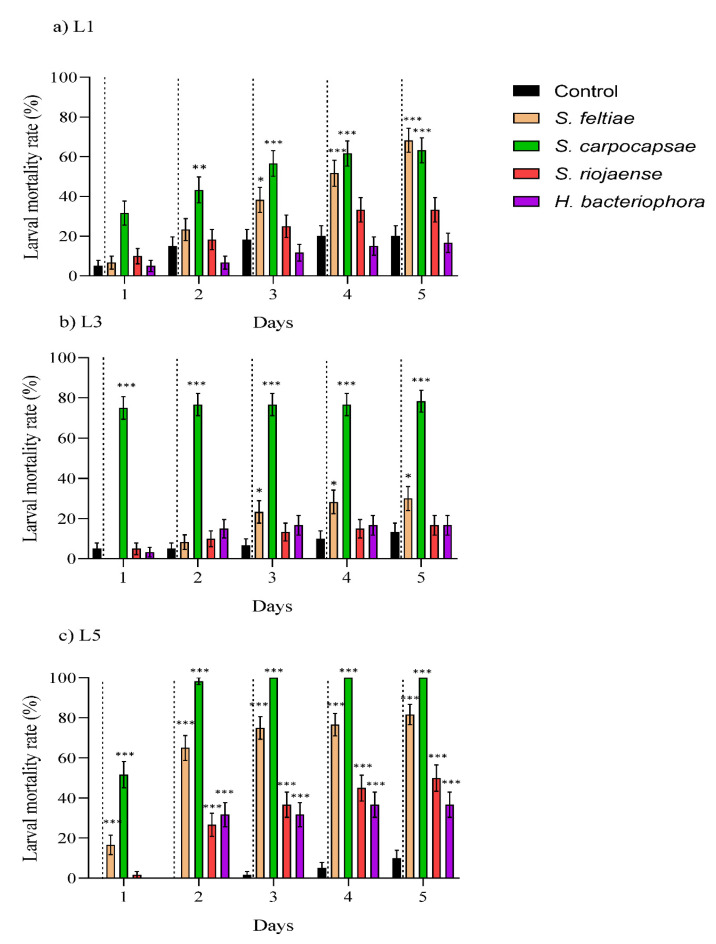
Virulence of the entomopathogenic nematodes species *Steinernema feltiae*, *S. carpocapsae*, *S. riojaense*, and *Heterorhabditis bacteriophora* against various larval instars the European grapevine moth, *Lobesia botrana*. (**a**) First instar (L1), (**b**) third instar (L3), and (**c**) fifth instar (L5). Data are presented in days (from 1 up to 5 days, x-axis) and measured as larval mortality rate (%) (y-axis). Asterisks indicate significant differences at *** *p* < 0.001, ** *p* < 0.01, * *p* < 0.05. Values are least-square means ± SE.

The bioassays for the larvicidal effects of the IJs against the L1, L3, and L5 EGVM instars showed that the EPN species *S. feltiae* and *S. carpocapsae* produced significantly higher mortality rates than the controls for all of the larval instars, while *S. riojaense* and *H. bacteriohora* only showed significant mortality rates against L5 (Figure 1; Supplementary Material, Table S3). The highest and fastest larval mortality rates were observed for *S. carpocapsae*, particularly against L3, reaching >75% mortality in 24 h (Figure 1b; Supplementary Material, Table S3). The concentration–mortality test for L5 showed that less than 1 IJ of *S. carpocapsae* was required for LC_50_, while 5 IJs was estimated to be necessary for *S. feltiae* (Table 3).

### 3.2. Larvicidal Effect by Bioactive Compounds Generated by the Symbiotic Bacteria

**Figure 2:**
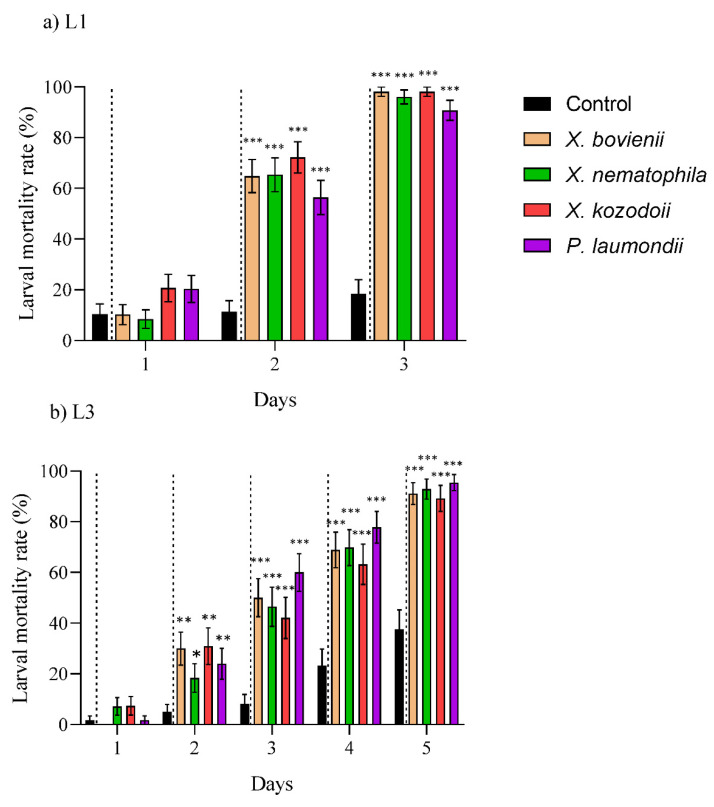
Toxic effect of the natural products produced by the symbiotic bacteria *Xenorhabdus bovienii*, *X. nematophila*, *X. kozodoii*, and *Photorhabdus laumondii* included in the cell-free supernatants tested against various larval instars of the European grapevine moth, *Lobesia botrana.* (**a**) First instar (L1) and (**b**) third instar (L3). Data are presented in days (from 1 up to 3 or 5 days, x-axis) and measured as larval mortality rate (%) (y-axis). Asterisks indicate significant differences at *** *p* < 0.001, ** *p* <0.01, * *p* < 0.05. Values are least-square means ± SE.

**Figure 3:**
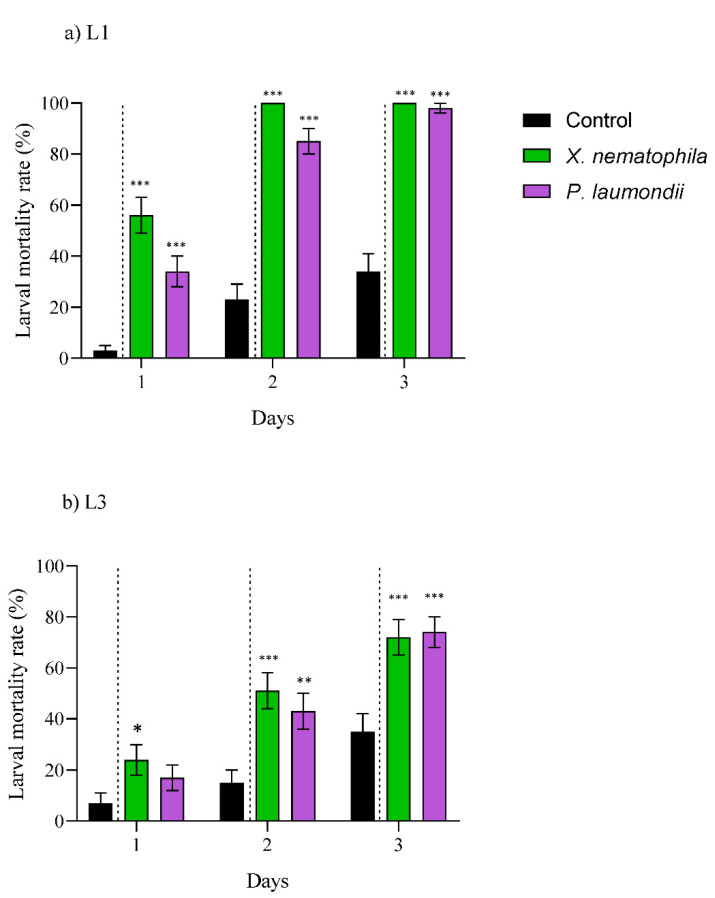
Toxic effect of the natural products produced by the symbiotic bacteria *Xenorhabdus nematophila* and *Photorhabdus laumondii* present in the unfiltered ferment (UF) against various larval instars the European grapevine moth, *Lobesia botrana.* (**a**) First instar (L1) and (**b**) third instar (L3). Data are presented in days (from 1 up to 3 days, x-axis) and measured as larval mortality rate (%) (y-axis). Asterisks indicate significant differences at *** *p* < 0.001, ** *p* < 0.01, * *p* < 0.05. Values are least-square means ± SE.

The CFS derived from the four symbiotic bacteria were toxic when ingested by both of the EGVM larval instars (Figure 2; Supplementary Material, Table S4). Against L1, the mortality rates exceeded 50% and 90% after two and three days, respectively (Figure 2a), while for L3, up to 4–5 days were needed to reach comparable numbers (Figure 2b). Similarly, the ingestion of UF products from both symbiotic bacteria were toxic against the L1 (over 80% in two days) and L3 (over 60% in three days) larval instars (Figure 3; Supplementary Material, Table S5).

### 3.3. Pupicidal Effect by the Entomopathogenic Nematodes

**Figure 4:**
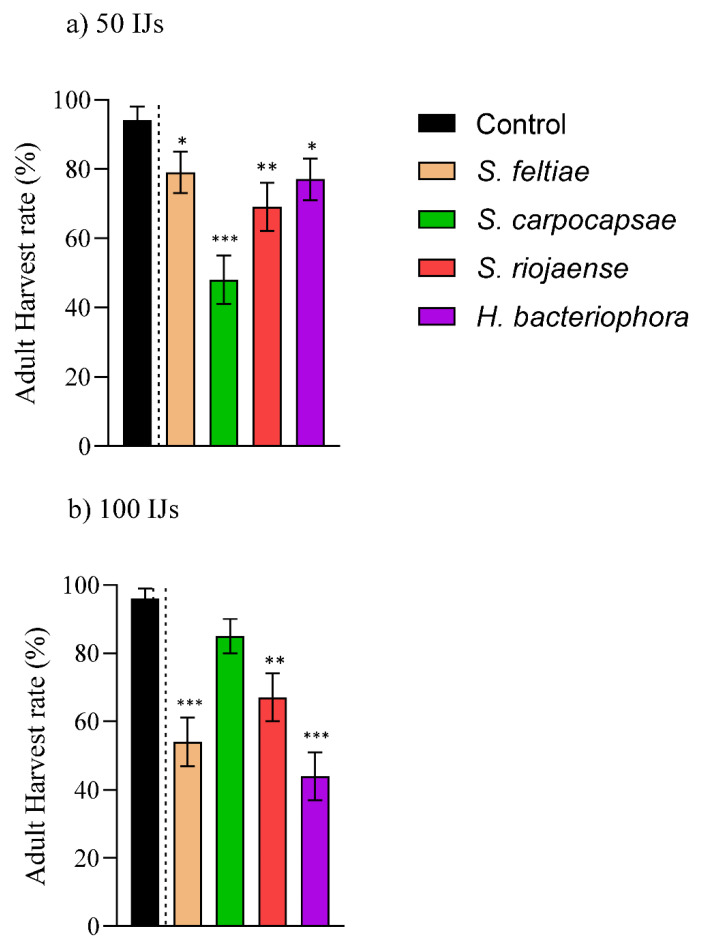
Virulence of infective juveniles (Ijs) of the *Steinernema feltiae, S. carpocapsae, S. riojaense,* and *Heterorhabditis bacteriophora* species against pupae of the European grapevine moth, *Lobesia botrana*. Concentration of (**a**) 50 IJs and (**b**) 100 IJs per pupa. Asterisks indicate significant differences at *** *p* < 0.001, ** *p* < 0.01, * *p* < 0.05. Values are least-square means ± SE.

For the 50 IJ applications, there were not significant differences compared to the controls for *S. feltiae*. However, the EGVM adult emergences were below 50% for *S. carpocapsae* (Figure 4a) only. Duplicating the concentration to 100 IJs per host, the EPN species *S. feltiae*, *S. riojaense*, and *H. bacteriophora* reduced the adult emergences to 46, 33 and 56%, respectively, while they did not improve the efficiency of *S. carpocapsae*, resulting in a higher adult emergence than the one observed at 50 IJs/cm^2^ (Figure 4b; Supplementary Material, Table S6).

## 4. Discussion

### 4.1. Entomopathogenic Nematodes as Biological Control Agents against Larvae and Pupae of the European Grapevine Moth

This study showed that the EPNs could be effective biological control agents against EGVM larvae and pupae in vineyards. In agreement with previous studies against other tortricid species, including *L. vanillana,* EPN virulence differed among nematode species [35,38,39,41,42]. As observed for *C. pomonella* [38], our *S. carpocapsae* population resulted in being the most virulent against the various larval and pupal stages. However, the virulence varied depending on the larval instar, with L1 being the least susceptible, which was probably due to size reasons and may have been too small for EPN. Bastidas et al. (2014) [43] showed that EPNs have limited efficacy against microarthropod hosts that are ~0.5 mm size. On average, *L. botrana* L1 is 0.9–1.5 mm long, while L3 and L5 are 4.5–5.0 and 10.0–11.0 mm, respectively. Consequently, the L1 can present smaller natural openings that limit colonization by IJs [43]. EPN species with a small-sized IJ such as *S. carpocapsae* can overcome this physical barrier [44]. In addition, L1 and L3 are instars that actively search for food and move intensely in the experimental arena. Hence, EPNs with an ambusher (*S. carpocapsae*) or intermediate (*S. feltiae*) searching behavior might be favored, while nematodes that are expected to display a cruiser behavior (*H. bacteriophora* and *S. riojaense*) can obtain limited results [45,46]. The reduced size of the host linked to the EPN cruiser behavior can explain the low larval mortality observed for *H. bacteriophora* and *S. riojaense,* only reaching ~40% mortality against L5 after five days of exposure. On the contrary, *S. carpocapsae* and *S. feltiae* registered 100% and 80% L5 mortality, respectively, at the same time exposure. Overall, the efficacy of our EPN populations at 48 IJs per host against *L. botrana* obtained similar results to those observed for two South African EPN species against *L. vanillana* but employed 100 IJs per host [39]. Indeed, the efficacy of these two EPN species in the 50% lethal concentration estimations against L5 EGVM is notorious. *S. carpocapsae* only required 0.3 Ijs/cm^2^, and *S. feltiae* only required 5.2 IJ/cm^2^ in only three days.

Although the pupal stage is less conductive for EPNs [35,39,41,42], our results showed that when employing high IJ concentrations, EGVM adult emergences can be significantly restricted. As for the larval stages, *S. carpocapsae* resulted in the most virulent species, reducing the adult emergence to below 50% when applied at the concentration of 50 IJs per host (~50% pupal mortality if corrected with the control emergence). On the other hand, the species *S. feltiae* and *H. bacteriophora* required double the concentration (100 IJs/host) to achieve similar values, while *S. riojanese,* which has a bigger IJ size [46], only registered ~65% emergence rates. The efficacy of various EPN species against pupa of *C. pomonella* using 50 IJs per host ranged from 20–75% in terms of pupal mortality [41], which is a similar pattern to the one observed for our populations at the same concentration (20–50% pupal mortality if converted from adult emergence). However, compared to the closely related species *L. vanillana,* with ~15% pupal mortality or less, depending on the EPN production system [39], the results obtained for *L. botrana* are promising. In addition, we observed that the presence of EPNs drove miniature EGVM adult emergences from the pupae (I. Vicente-Díez, personal observation). A recent study has shown that the presence of EPNs can alter developmental times and changes in the risk of death of the non-susceptible pupal stage of *Delia antiqua* (Diptera: Anthomyiidae) [47]. As such, this possible alteration in size as well as potential alterations in other metabolic parameters might be of interest in the context of the preventive and biological control of EGVMs. Further research is required to confirm and characterize this non-lethal effect.

### 4.2. Natural Products Derived from Xenorhabdus and Photorhabdus Have Toxic Effect on Larvae

The CFS and UF products obtained from the symbiotic EPN bacteria exhibited high toxicity against the L1 and L3 EGVM instars, arising as novel biotech tools against this particular pest. In the evaluation of the effect against various pests and pathogens, CFS was the most prevalent system [27,40,48,49]. However, Bussaman et al. (2009) [50] also showed the potential of UF against the mushroom mite *Luciaphorus perniciosus* (Acari: Pygmephoridae), also reporting the non-lethal effect of reducing pest fecundity. Similarly, Steyn et al. (2021) [42] showed that the UF application caused significantly higher egg mortality on *T. leucotreta* than in the control treatment, although the mechanism behind this effect is unknown. Still, to the best of our knowledge, no previous studies analyzed the efficacy of CFS and UF products against the same target. We have demonstrated for the first time that the use of UF products derived from the bacterial species *X. nematophila* and *P. laumondii* can lead to a faster and stronger effect against L1 and L3 instars than their CFS. Today, there are different commercial biopesticides that are classified according to the active substance: (*i*) micro-organisms, (*ii*) biochemicals, and (*iii*) semiochemicals [51]. Despite the massive range of possibilities that exist in the development of this new-biotechnological control approach, *B. thuringiensis* (Table 1) products developed for control of agricultural insect pests (e.g., EGVM) are the most widely spread, representing approximately 95% of micro-organisms that are used for pest control [52]. Research on *Xenorhabdus* and *Photorhabdus* based products increase their range of action as biopesticides, biofungicides, or bioacaricials [27,49,53]. Their demonstrated oral toxicity against larval instars of EGVM makes them a potential novel biopesticide, with the UF products as a promising area of exploration for new biocompounds and activities.

## 5. Conclusions

The principal challenges facing all agriculture and especially the grape industry are intensive pesticide use, invasion by new pests/diseases, and climate change [11]. Enhancing good practices for pest, disease, and disease vector management would help address these challenges [40,54]. By maintaining the biodiversity of the vineyard agroecosystem, natural enemies of arthropods can contribute to crop protection [55,56,57]. In this study, we compared different EPN populations as facultative biological control agents against larval and pupal instars of the key grape pest, EGVM. It is likely that EPNs might control other stages such as adults and eggs, as shown for other tortricids [41,42]. In addition, future viticulture reclaims innovative biotechnical tools that maintain annual crop production while the progressive reduction of chemical supplies becomes legislated. The use of microorganisms such as *Xenorhabdus* spp. and *Photorhabdus* spp. offer promising and environmentally friendly strategies for conventional and organic viticulture worldwide [3,40,58]. Advances in the aerial application of EPNs, the characterization of specific active compounds, and the evaluation of their efficacy and potential risk for other biocontrol agents and the environment will allow the adoption of this technology by growers in a near future.

## Figures and Tables

**Table 1:** Overview of biological control agents and biotechnical control tools against grapevine moths in viticulture and the facultative use of entomopathogenic nematodes and their symbiotic bacteria-based products.

				Principal Target	References
Biological control agents	Entomopathogenic fungi		*Beauviera bassiana*	Pupae	López Plantey et al., 2019 [15]
			*Metarhizium* spp. (*M. robertsii*; *M. anisopliae*)	Larvae	López Plantey et al., 2019; Sammaritano et al., 2018 [15,21]
	Bacterias		*B. thuringiensis*	Larvae Adults	Ioriatti et al., 2011 [20]
	Artropods	Predator	*Chrysoperla sp*.	Eggs Pupae	Castex et al., 2018 [16]
		Parasitoids	*Trichogramma* sp.	Eggs	Pérez Moreno et al., 2000 [22]
			*Dibrachis* sp.	Pupae	
	Entomopathogenic nematodes		*Steinernema* sp. *Heterohabditis* sp.	Larvae Pupae	Current research
Biotechnical control tools	Pheromone-mediated Malting Disruption (MD)			Adults	Ioriatti et al., 2011; Shapira et al., 2018 [19,20]
	Insecticidal-plants extracts		*Bifora radians*	Larvae	Gökçe et al., 2011 [23]
	Insecticidal-bacterium based product		*Bacillus thuringiensis*	Larvae	Ifoulis and Savopoulou-Soultani, 2004 [24]
			*Xhenorhabdus* sp. *Photorhabdus* sp.	Larvae	Current reserch

**Table 2:** *Steinernema* and *Heterorhabditis* species and their symbiotic bacteria species (*Xenorhaddus* and *Photorhabdus*) tested for their effects as biocontrol agent against *Lobesia botrana*.

Entomopathogenic Nematodes Species	Population	ITS-Gen Bank Accession Number	Bacterial Species	ITS-Gen Bank Accession Number
*Steinernema feltiae*	RM-107	MW480131	*Xenorhabdus bovienii*	MW467374
*Steinernema carpocapsae*	All	MW574913	*Xenorhabdus nematophila*	MW574906
*Steinernema riojaense*	RM-30	MK503133	*Xenorhabdus kozodoii*	MW467375
*Heterorhabditis bacteriophora*	RM-102	MW480132	*Photorabhdus laumondii* subsp. *laumondii*	MW574908

**Table 3:** Lethal concentration (LC) responses against L5 larval stages of the European grapevine moth (EGVM), *Lobesia botrana* estimated for the entomopathogenic nematodes species *Steinernema feltiae* (Sfe) and *Steinernema carpocapsae* (Sca).

Day	Treatment	Tested Stage of EGVM	Number of Insect Tested	Slope ± SE	LC_50_ (95% Confidence Intervals)	LC_90_ (95% Confidence Intervals)	*X* ^2^
3	Sfe	L5	300	0.918 ± 0.222	5.229 (3.469–10.297)	130.362 (38.089–3766.676)	1.383
	Sca	L5	300	2.112 ± 0.623	0.352 (0.047–0.629)	1.424 (0.954–2.074)	1.398
4	Sfe	L5	300	1.001 ± 0.223	3. 692 (2.499–5.821)	70.318 (26.189–801.379)	4.395
	Sca	L5	300	3.248 ± 1.073	0.530 (0.110–0.769)	1.315 (1.024–1.887)	0.065

## Data Availability

Data provided as requested.

## Supplementary Materials

The following are available online at https://www.mdpi.com/article/10.3390/insects12111033/s1, Table S1: Ingredients and quantity of the artificial diet for *Lobesia botrana* larvae.; Table S2: Ingredients and quantity of the artificial diet for *Galleria mellonella* larvae.; Table S3: Results from generalized linear mixed models testing within pair-treatment comparisons of control and each entomopathogenic nematode tested.; Table S4: Results from generalized linear mixed models testing within pair-treatment comparisons of control and each bacterial-cell free supernatant tested.; Table S5: Results from generalized linear mixed models testing within pair-treatment comparisons of control and each bacterial unfiltered-ferment tested.; Table S6: Results from generalized linear mixed models testing within pair-treatment comparisons of control and each entomopathogenic nematode tested at 10 days.

## References

[1] Santos J.A., Fraga H., Malheiro A.C., Moutinho-Pereira J., Dinis L.-T., Correia C., Moriondo M., Leolini L., Dibari C., Costafreda-Aumedes S. (2020). A Review of the Potential Climate Change Impacts and Adaptation Options for European Viticulture. Appl. Sci..

[2] Pertot I., Caffi T., Rossi V., Mugnai L., Hoffmann C., Grando M.S., Gary C., Lafond D., Duso C., Thiery D. (2017). A critical review of plant protection tools for reducing pesticide use on grapevine and new perspectives for the implementation of IPM in viticulture. Crop Prot..

[3] Provost C., Pedneault K. (2016). The organic vineyard as a balanced ecosystem: Improved organic grape management and impacts on wine quality. Sci. Hortic..

[4] Martín Gil Á., Ramos Sáez de Ojer J.L., Pérez M.R. (2014). Guía de Gestión Integrada de Plagas: Uva de Transformación.

[5] Zehnder G., Gurr G.M., Kühne S., Wade M.R., Wratten S.D., Wyss E. (2007). Arthropod pest management in organic crops. Annu. Rev. Entomol..

[6] Jacometti M.A., Wratten S.D., Walter M. (2010). Review: Alternatives to synthetic fungicides for *Botrytis cinerea* management in vineyards. Aust. J. Grape Wine Res..

[7] Gonzalez M. (2010). Lobesia botrana: Polilla de la uva. Enología.

[8] Varela L.G., Lucchi A., Bagnoli B., Nicolini G., Ioriatti C. (2013). Impacts of standard wine-making process on the survival of *Lobesia botrana* larvae (Lepidoptera: Tortricidae) in infested grape clusters. J. Econ. Entomol..

[9] Gilligan T.M., Epstein M.E., Passoa S.C., Powell J.A., Sage O.C., Brown J.W. (2011). Discovery of *Lobesia botrana* ([Denis & Schiffermller]) in California: An invasive species new to North America (Lepidoptera: Tortricidae). Proc. Entomol. Soc. Washingt..

[10] Rank A., Ramos R.S., da Silva R.S., Soares J.R.S., Picanço M.C., Fidelis E.G. (2020). Risk of the introduction of *Lobesia botrana* in suitable areas for *Vitis vinifera*. J. Pest Sci..

[11] Gutierrez A.P., Ponti L., Gilioli G., Baumgärtner J. (2018). Climate warming effects on grape and grapevine moth (*Lobesia botrana*) in the Palearctic region. Agric. For. Entomol..

[12] Martín-Vertedor D., Ferrero-García J.J., Torres-Vila L.M. (2010). Global warming affects phenology and voltinism of *Lobesia botrana* in Spain. Agric. For. Entomol..

[13] Reis S., Martins J., Gonçalves F., Carlos C., Santos A.J. (2021). European grapevine moth in the Douro region: Voltinism and climatic scenarios. OENO One.

[14] Mondani L., Palumbo R., Tsitsigiannis D., Perdikis D., Mazzoni E., Battilani P. (2020). Pest Management and Ochratoxin A Contamination in Grapes: A review. Toxins.

[15] López Plantey R., Papura D., Couture C., Thiéry D., Pizzuolo P.H., Bertoldi M.V., Lucero G.S. (2019). Characterization of entomopathogenic fungi from vineyards in Argentina with potential as biological control agents against the European grapevine moth *Lobesia botrana*. BioControl.

[16] Castex V., Beniston M., Calanca P., Fleury D., Moreau J. (2018). Pest management under climate change: The importance of understanding tritrophic relations. Sci. Total Environ..

[17] Lacey L.A., Grzywacz D., Shapiro-Ilan D.I., Frutos R., Brownbridge M., Goettel M.S. (2015). Insect pathogens as biological control agents: Back to the future. J. Invertebr. Pathol..

[18] Yoshida S., Koitabashi M., Yaginuma D., Anzai M., Fukuda M. (2019). Potential of bioinsecticidal *Bacillus thuringiensis* inoculum to suppress gray mold in tomato based on induced systemic resistance. J. Phytopathol..

[19] Shapira I., Keasar T., Harari A.R., Gavish-Regev E., Kishinevsky M., Steinitz H., Sofer-Arad C., Tomer M., Avraham A., Sharon R. (2018). Does mating disruption of *Planococcus ficus* and *Lobesia botrana* affect the diversity, abundance and composition of natural enemies in Israeli vineyards?. Pest Manag. Sci..

[20] Ioriatti C., Anfora G., Tasin M., De Cristofaro A., Witzgall P., Lucchi A. (2011). Chemical ecology and management of *Lobesia botrana* (Lepidoptera: Tortricidae). J. Econ. Entomol..

[21] Sammaritano J.A., Deymié M., Herrera M., Vazquez F., Cuthbertson A.G.S., López-Lastra C., Lechner B. (2018). The entomopathogenic fungus, *Metarhizium anisopliae* for the european grapevine moth, *Lobesia botrana* Den. & Schiff. (Lepidoptera: Tortricidae) and its effect to the phytopathogenic fungus, *Botrytis cinerea*. Egypt. J. Biol. Pest Control.

[22] Pérez Moreno I., Marco Mancebón V., Sáenz de Cabezón F. (2000). Evaluación del parasitismo natural sobre crisálidas hibernants de polilla del racimo (*Lobesia botrana* Den. y Schiff.) en viñedos de La Rioja. Boletín Sanid. Veg. Plagas.

[23] Gökçe A., Isaacs R., Whalon M.E. (2011). Ovicidal, larvicidal and anti-ovipositional activities of *Bifora radians* and other plant extracts on the grape berry moth *Paralobesia viteana* (Clemens). J. Pest Sci..

[24] Ifoulis A.A., Savopoulou-Soultani M. (2004). Biological control of *Lobesia botrana* (Lepidoptera: Tortricidae) larvae by using different formulations of *Bacillus thuringiensis* in 11 vine cultivars under field conditions. J. Econ. Entomol..

[25] Shapiro-Ilan D.I., Han R., Dolinksi C. (2012). Entomopathogenic nematode production and application technology. J. Nematol..

[26] Griffin C.T. (2015). Behaviour and population dynamics of entomopathogenic nematodes following application. Nematode Pathogenesis of Insects and Other Pests.

[27] Da Silva W.J., Pilz-Júnior H.L., Heermann R., Da Silva O.S. (2020). The great potential of entomopathogenic bacteria *Xenorhabdus* and *Photorhabdus* for mosquito control: A review. Parasites Vectors.

[28] Karthik Raja R., Arun A., Touray M., Hazal Gulsen S., Cimen H., Gulcu B., Hazir C., Aiswarya D., Ulug D., Cakmak I. (2021). Antagonists and defense mechanisms of entomopathogenic nematodes and their mutualistic bacteria. Biol. Control.

[29] Boemare N., Gaugle R. (2002). Biology, taxonomy and systematics of *Xenorhabdus* and *Photorhabdus*. Entomopathogenic Nematology.

[30] Adams B.J., Fodor A., Koppenhöfer H.S., Stackebrandt E., Patricia Stock S., Klein M.G. (2006). Biodiversity and systematics of nematode-bacterium entomopathogens. Biol. Control.

[31] Dillman A.R., Chaston J.M., Adams B.J., Ciche T.A., Goodrich-Blair H., Stock S.P., Sternberg P.W. (2012). An entomopathogenic nematode by any other name. PLoS Pathog..

[32] Campos-Herrera R., Vicente-Díez I., Blanco-Pérez R., Chelkha M., del Mar Gonzalez-Trujillo M., Puelles M., Čepulitè R., Pou A. (2021). Positioning entomopathogenic nematodes for the future viticulture: Exploring their use against biotic threats and as bioindicators of soil health. Turk. J. Zool..

[33] Shapiro-Ilan D., Dolinksi C., Campos-Herrera R. (2015). Entomopathogenic Nematode Application Technology. Nematode Pathogenesis of Insects and Other Pests: Ecology and Applied Technologies for Sustainable Plant and Crop Protection.

[34] Manrakhan A., Daneel J.H., Moore S.D. (2014). The impact of naturally occurring entomopathogenic nematodes on false codling moth, *Thaumatotibia leucotreta* (Lepidoptera: Tortricidae), in citrus orchards. Biocontrol Sci. Technol..

[35] Odendaal D., Addison M.F., Malan A.P. (2016). Control of diapausing codling moth, *Cydia pomonella* (Lepidoptera: Tortricidae) in wooden fruit bins, using entomopathogenic nematodes (*Heterorhabditidae* and *Steinernematidae*). Biocontrol Sci. Technol..

[36] de Waal J.Y., Addison M.F., Malan A.P. (2018). Potential of *Heterorhabditis zealandica* (Rhabditida: Heterorhabditidae) for the control of codling moth, *Cydia pomonella* (Lepidoptera: Tortricidae) in semi-field trials under South African conditions. Int. J. Pest Manag..

[37] Malan A.P., Diest J.I.V., Moore S.D., Addison P. (2018). Control Options for False Codling Moth, *Thaumatotibia leucotreta* (Lepidoptera: Tortricidae), in South Africa, with Emphasis on the Potential Use of Entomopathogenic Nematodes and Fungi. Afr. Entomol..

[38] Yağci M., Özdem A., Erdoğuş F.D., Ayan E. (2021). Efficiency of entomopathogenic nematodes (Rhabditida: *Heterorhabditidae* and *Steinernematidae*) on the codling moth (*Cydia pomonella* L.) (Lepidoptera: Tortricidae) under controlled conditions. Egypt. J. Biol. Pest Control.

[39] du Preez F., Malan A.P., Addison P. (2021). Potential of in vivo- and in vitro-cultured entomopathogenic nematodes to infect *Lobesia vanillana* (Lepidoptera: Tortricidae) under laboratory conditions. PLoS ONE.

[40] Vicente-Díez I., Blanco-Pérez R., del Mar Gonzalez-Trujillo M., Pou A., Campos-Herrera R. (2021). Insecticidal Effect of Entomopathogenic Nematodes and the Cell-Free Supernatant from Their Symbiotic Bacteria against *Philaenus spumarius* (Hemiptera: Aphrophoridae) Nymphs. Insects.

[41] Malan A.P., Knoetze R., Moore S.D. (2011). Isolation and identification of entomopathogenic nematodes from citrus orchards in South Africa and their biocontrol potential against false codling moth. J. Invertebr. Pathol..

[42] Steyn V.M., Malan A.P., Addison P. (2021). Efficacy of entomopathogens against *Thaumatotibia leucotreta* under laboratory conditions. Entomol. Exp. Appl..

[43] Bastidas B., Portillo E., San-Blas E. (2014). Size does matter: The life cycle of *Steinernema* spp. in micro-insect hosts. J. Invertebr. Pathol..

[44] Stock S.P., Campos-Herrera R. (2015). Diversity, biology and evolutionary relationships. Nematode Pathogenesis of Insects and Other Pests.

[45] Campbell J.F., Lewis E.E., Stock S.P., Nadler S., Kaya H.K. (2003). Evolution of host search strategies in entomopathogenic nematodes. J. Nematol..

[46] Půža V., Campos-Herrera R., Blanco-Pérez R., Jakubíková H., Vicente-Díez I., Nermut’ J. (2020). *Steinernema riojaense* n. sp., a new entomopathogenic nematode (Nematoda: Steinernematidae) from Spain. Nematology.

[47] Filgueiras C.C., Willett D.S. (2021). Non-lethal effects of entomopathogenic nematode infection. Sci. Rep..

[48] Chacón-Orozco J.G., Bueno C.J., Shapiro-Ilan D.I., Hazir S., Leite L.G., Harakava R. (2020). Antifungal activity of *Xenorhabdus* spp. and *Photorhabdus* spp. against the soybean pathogenic *Sclerotinia sclerotiorum*. Sci. Rep..

[49] Eroglu C., Cimen H., Ulug D., Karagoz M., Hazir S., Cakmak I. (2019). Acaricidal effect of cell-free supernatants from *Xenorhabdus* and *Photorhabdus* bacteria against *Tetranychus urticae* (Acari: Tetranychidae). J. Invertebr. Pathol..

[50] Bussaman P., Sobanboa S., Grewal P.S., Chandrapatya A. (2009). Pathogenicity of additional strains of *Photorhabdus* and *Xenorhabdus* (Enterobacteriaceae) to the mushroom mite *Luciaphorus perniciosus* (Acari: Pygmephoridae). Appl. Entomol. Zool..

[51] Chandler D., Bailey A.S., Mark Tatchell G., Davidson G., Greaves J., Grant W.P. (2011). The development, regulation and use of biopesticides for integrated pest management. Philos. Trans. R. Soc. B Biol. Sci..

[52] Schünemann R., Knaak N., Fiuza L.M. (2014). Mode of Action and Specificity of *Bacillus thuringiensis* Toxins in the Control of Caterpillars and Stink Bugs in Soybean Culture. ISRN Microbiol..

[53] Orozco R.A., Molnár I., Bode H., Stock S.P. (2016). Bioprospecting for secondary metabolites in the entomopathogenic bacterium *Photorhabdus luminescens* subsp. sonorensis. J. Invertebr. Pathol..

[54] Crowder D.W., Jabbour R. (2014). Relationships between biodiversity and biological control in agroecosystems: Current status and future challenges. Biol. Control.

[55] Blanco-Pérez R., Sáenz-Romo M.G., Vicente-Díez I., Ibáñez-Pascual S., Martínez-Villar E., Marco-Mancebón V.S., Pérez-Moreno I., Campos-Herrera R. (2020). Impact of vineyard ground cover management on the occurrence and activity of entomopathogenic nematodes and associated soil organisms. Agric. Ecosyst. Environ..

[56] Karimi B., Cahurel J.Y., Gontier L., Charlier L., Chovelon M., Mahé H., Ranjard L. (2020). A meta-analysis of the ecotoxicological impact of viticultural practices on soil biodiversity. Environ. Chem. Lett..

[57] Sáenz-Romo M.G., Veas-Bernal A., Martínez-García H., Campos-Herrera R., Ibáñez-Pascual S., Martínez-Villar E., Pérez-Moreno I., Marco-Mancebón V.S. (2019). Ground cover management in a Mediterranean vineyard: Impact on insect abundance and diversity. Agric. Ecosyst. Environ..

[58] Mnif I., Ghribi D. (2015). Potential of bacterial derived biopesticides in pest management. Crop Prot..

