# Supplementary material for: Exploring the Use of Entomopathogenic Nematodes and the Natural Products Derived from Their Symbiotic Bacteria to Control the Grapevine Moth, Lobesia botrana (Lepidoptera: Tortricidae)
Source: Insects. 2021 Nov 17;12(11):1033. doi: 10.3390/insects12111033 (PMC8622629; doi:10.3390/insects12111033)
Supplement: Supplementary file 1 [file insects-12-01033-s001.zip › insects-1440351-supplementary.pdf]

Supplementary Material 1.

Table S1. Ingredients and quantity of the artificial diet for *Lobesia botrana* larvae.

Ingredients	Quantity
Main ingredients	
Corn flour	65 g
Wheat germ	78 g
Beer yeast	65 g
Corn oil	2 ml
Vitamin source	
Ascorbic acid	6,4 g
Preservative	
Benzoic acid	2 g
p-Hydroxybenzoic acid esters (Nipagina)	2 g
Chlortetracycline hydrochloride (Potency 900µg/mg)	0,75 g
Fumagilina 2% (Nosapiol-B)	0,3 g
Agar	20 g
Water	1.1000 ml

Table S2. Ingredients and quantity of the artificial diet for *Galleria mellonella* larvae.

Ingredients	Quantity
Dry dog food	932 g
Water	532 g
Honey	267 g
Glycerine	267 g
Wheat bran	500 g

Supplementary Material 2.

Table S3. Results from generalized linear mixed models testing within pair-treatment comparisons of control and each entomopathogenic nematode tested.

Asterisks indicate significant at ***P<0.001, **P<0.01, *P<0.05, n.s., not significant.

	L1										L3										L5									
	D1		D2		D3		D4		D5		D1		D2		D3		D4		D5		D1		D2		D3		D4		D5	
	X^2	P	X^2	P	X^2	P	X^2	P	X^2	P	X^2	P	X^2	P	X^2	P	X^2	P	X^2	P	X^2	P	X^2	P	X^2	P	X^2	P	X^2	P
S. feltiae	0.151	n.s.	1.342	n.s.	5.88	*	12.96	***	27.834	***	0	n.s.	0.533	n.s.	6.37	*	6.418	*	4.876	*	12	***	111.429	***	158.256	***	136.123	***	128.552	***
S. carpocapsae	13.257	n.s.	11.51	**	18.527	***	21.224	***	22.791	***	51.051	***	52.989	***	52.943	***	50.146	***	48.232	***	64.138	***	3540	***	3540	***	1140	***	540	***
S. riojaense	1.072	n.s.	0.24	n.s.	0.785	n.s.	2.721	n.s.	2.721	n.s.	0	n.s.	1.072	n.s.	1.471	n.s.	0.682	n.s.	0.261	n.s.	1.017	n.s.	21.818	***	29.564	***	32.542	***	28.235	***
H. bacteriphora	0	n.s.	2.135	n.s.	1.043	n.s.	0.519	n.s.	0.223	n.s.	0.208	n.s.	3.261	n.s.	2.874	n.s.	1.15	n.s.	0.261	n.s.	0	n.s.	0	n.s.	14.544	***	16.718	***	11.646	**

Table S4. Results from generalized linear mixed models testing within pair-treatment comparisons of control and each bacterial-cell free supernatant tested. Asterisks indicate significant at ***P<0.001, **P<0.01, *P<0.05, n.s., not significant.

	L1						L3									
	D1		D2		D3		D1		D2		D3		D4		D5	
	X^2	P	X^2	P	X^2	P	X^2	P	X^2	P	X^2	P	X^2	P	X^2	P
X. bovienii	0.001	n.s.	30.8	***	43.02	***	0	n.s.	11.287	**	19.595	***	18.11	***	25.179	***
X. nematophila	0.12	n.s.	30.903	***	48.486	***	1.927	n.s.	4.573	*	16.815	***	18.381	***	25.322	***
X. kozodoii	2.354	n.s.	38.325	***	43.102	***	2.032	n.s.	11.212	**	13.397	***	12.993	***	20.796	***
P. laumondii	2.234	n.s.	23.385	***	49.618	***	0	n.s.	7.686	**	26.876	***	25.469	***	27.141	***

Table S5. Results from generalized linear mixed models testing within pair-treatment comparisons of control and each bacterial unfiltered-ferment tested.

Asterisks indicate significant at ***P<0.001, **P<0.01, *P<0.05, n.s., not significant.

	L1						L3					
	D1		D2		D3		D1		D2		D3	
	X^2	P	X^2	P	X^2	P	X^2	P	X^2	P	X^2	P
X. nematophila	31.268	***	41.257	***	32.625	***	5.516	*	13.586	***	12.572	***
P. laumondii	15.593	***	41.441	***	28.88	***	2.564	n.s.	8.82	**	14.075	***

Table S6. Results from generalized linear mixed models testing within pair-treatment comparisons of control and each entomopathogenic nematode tested at 10 days. Concentration of 50 IJs and 100 IJs per pupa Asterisks indicate significant at ***P<0.001, **P<0.01, *P<0.05, n.s., not significant.

	50 IJs		100 IJs	
	X^2	P	X^2	P
S. feltiae	4.255	*	19.225	***
S. carpocapsae	22.179	***	2.97	n.s.
S. riojaense	9.286	**	12.101	**
H. bacteriophora	5.2	*	25.882	***
